# Ecological signature on the epidemiological dynamics of severe fever with thrombocytopenia syndrome

**DOI:** 10.1371/journal.pntd.0014408

**Published:** 2026-06-08

**Authors:** Zhe Lou, Jing Lu, Shuyi Liang, Wei Zhao, Ling Huang, Haowei Wang, Xiang Li, Jianli Hu, Ruiyun Li

**Affiliations:** 1 School of Public Health, Nanjing Medical University, Nanjing, China; 2 Department of Acute infectious Diseases Control and Prevention, Jiangsu Provincial Center for Disease Control and Prevention, Nanjing, China; 3 College of Urban and Environmental Sciences, Peking University, Beijing, China; 4 School of Public Health, Imperial College London, London, United Kingdom; 5 MRC Centre for Global Infectious Disease Analysis and Abdul Latif Jameel Institute for Disease and Emergency Analytics, Imperial College London, London, United Kingdom; 6 Jiangsu Center for Collaborative Innovation in Geographical Information Resource Development and Application, Nanjing Normal University, Nanjing, China; Hainan University, CHINA

## Abstract

**Background:**

Severe fever with thrombocytopenia syndrome (SFTS) is prioritized as an emerging tick-borne disease, posing a continuing threat to human populations. Existing evidence has shown that tick-to-human transmission is the primary route of human infection. However, this insight has not been consistently incorporated into studies examining the ecological dependence of vectored disease dynamics.

**Methods:**

We employ an eco-epidemiological model to assess tick abundance in response to the natural environment and its contribution to SFTS transmission. Our statistical model, which integrates demographic, ecological, and behavioural factors, investigates how vector abundance impacts case fatality risk.

**Results:**

Our findings identified a shift in peak incidence from July to May among endemic counties since 2021. Additionally, we show that local climate conditions influence SFTS dynamics by modulating local vector populations, revealing clear spatial heterogeneity in transmission potential across endemic counties. Further investigations suggest that the odds of death are higher for patients living in the areas with higher tick abundance.

**Conclusions:**

These insights emphasize the profound influence of ecological factors on disease dynamics and severity. Therefore, understanding the interrelation between climate and vector population dynamics is crucial for both research and policymaking related to climate-sensitive vectored diseases.

## Author summary

Severe fever with thrombocytopenia syndrome (SFTS) is an emerging tick-borne disease that continues to threaten rural populations, yet ecological drivers of transmission and disease severity are often not explicitly linked to tick dynamics. In this study, we combined long-term tick surveillance, climate data, and human case records in Jiangsu Province, China, using an eco-epidemiological modelling framework that connects weather conditions to estimated tick abundance and then to human infections. We identified an important recent change in seasonality, with the peak of reported SFTS cases shifting from July to May in endemic counties since 2021. Our analyses suggest that local climate conditions shape SFTS dynamics by modulating local tick populations, producing clear geographic differences in transmission potential across counties. We also found that patients living in areas with higher estimated tick abundance had a higher risk of death after infection, even after accounting for demographic and behavioural factors. These findings highlight the value of integrating climate-informed vector indicators into surveillance and risk assessment to support earlier warnings and more targeted prevention for climate-sensitive tick-borne diseases.

## Introduction

Severe fever with thrombocytopenia syndrome (SFTS) is a tick-borne disease caused by SFTS virus, a newly identified phlebovirus in the family *Bunyaviridae* [1]. Cases with SFTS-like clinical manifestations, initially described as fever, thrombocytopenia and leukopenia syndrome, had been reported in Henan Province since 2007, while the causative virus was first identified during investigations of SFTS cases in Hubei and Henan provinces in 2009 [1,2]. ‌‌SFTS was subsequently confirmed in multiple provinces across central and northeastern China. The high case-fatality rate (CFR), which reached up to 30% in the early stage of the epidemic, together with the expanding endemic areas of SFTS, has contributed to its persistent public health threats since the identification of SFTSV [1,2]. Human cases are associated with wide clinical manifestations, varying from mild symptoms to life-threatening illness or death [3]. In view of continuing epidemic threats and insufficient preventive and curative solutions, SFTS is increasingly recognized as a prioritized emerging disease with the potential to generate public health emergencies [4]. This motivates researchers to support the urgently needed assessment of epidemic risks and ultimately unbiased intervention decisions.

As with other zoonotic diseases, SFTS risk assessment relies primarily on investigating how the ecological contexts modulate the transmission potential and severity of the disease [5,6]. It is widely recognized that tick-to-human transmission is the primary route of human infection with SFTS virus. Although the complete ecological cycle of SFTSV has not yet been fully elucidated, accumulating evidence identifies *Haemaphysalis longicornis* as the principal and confirmed vector, based on its high abundance in endemic areas and the frequent detection of viral RNA in field-collected specimens [1,3]. In addition, several other tick species have been proposed as potential contributors to SFTSV transmission, suggesting a more complex vector system that may vary across ecological settings. Vertebrate animals are thought to act mainly as amplifying hosts rather than true reservoirs, highlighting the central role of ticks in shaping human exposure risk [2,7,8]. The interplay of natural environment and tick population dynamics may shape the diverse ecological landscapes of local epidemic risk [9]. Crucially, meteorological conditions can shape local ecological contexts by influencing tick survival, development, and seasonal activity, thereby modulating opportunities for tick-human contact. Previous studies have shown that temperature, precipitation, and vegetation-related environmental factors are closely associated with the spatial distribution of SFTS risk [10,11]. Consequently, higher infection risks are frequently observed in mountainous or hilly rural areas where forested or shrub-dominated landscapes provide suitable habitats for ticks and where agricultural activities increase human exposure to tick bites [10–13]. However, existing epidemiological evidence has not consistently integrated ecological signature related to climate-associated variation in vector abundance and environmental context with the inference of SFTS dynamics and its severity.

The fundamental ecological dependence of vectored disease dynamics introduces an urgent need to contextualise mechanistic dynamics of SFTS in local ecological systems. Our recent research innovations have contributed to the development of eco-epidemiological models [5,6] that integrate natural environment, vector abundance and human cases. The interconnected nature of these models suggested that disease dynamics may be highly dependent on local vector dynamics, which, in turn, can be influenced by local climate conditions. Consequently, these models facilitate the concurrent analysis of vector responses to the natural environment and their impact on disease dynamics.

We ground our study in the context of the most recent increase in SFTS incidence and case fatality in Jiangsu Province, China [14,15]. We characterize the evolving epidemic profile of SFTS along two key dimensions: the spatiotemporal diffusion of infections and annual CFR. This perspective enables the identification of endemic counties, changes in severity and shifts in seasonality of the disease. We subsequently focus on an eco-epidemiological modelling framework to assess the dynamic risk of SFTS across these endemic counties. Within this framework, climate-based vectored dynamics are used as a proxy for the transmission, linking SFTS ecology with its epidemiological dynamics. Specifically, we test the hypothesis that (1) climate-associated increases in estimated tick abundance are associated with higher transmission potential ($R_{e}$) across endemic counties, and (2) variation in estimated vector abundance is independently associated with differences in case fatality risk among reported SFTS cases after accounting for demographic, ecological and behavioural factors. The findings of our study highlight the profound influence of ecological contexts on disease dynamics and severity, offering valuable insights applicable to a broad spectrum of zoonotic diseases.

## Methods

**Ethics statement:** This study was approved by the Ethics Committee of Jiangsu Provincial Center for Disease Prevention and Control, which waived the requirement for informed consent due to the retrospective nature of the study and the use of anonymized data. All methods were performed in accordance with the relevant guidelines and regulations. All experimental protocols were approved by the aforementioned institutional committee.

### Surveillance of ticks

Ticks surveillance was directed and supervised by the provincial and local centers of disease control and prevetion. Focusing on the tick activity season, tick samples were collected from March through October across multiple surveillance sites covering all 13 cities in Jiangsu Province. Tick surveillance data were collected between 2019 and 2022. Tick collection was performed following the standardized protocol of the Jiangsu Provincial SFTS surveillance programme (2023). Specifically, sampling was conducted between 6:00–8:00 am for two sequential days in the first week of each month. Tick flags (1 m^2^ white cotton flannel cloth) are the primary tools for tick collection which were moved from the surface of grass or shrubs. The flag was dragged or swept evenly across selected sampling plots, with inspection stops every 10 m to examine attached ticks. Tick density was quantified as a density index which is defined as the number of ticks captured per person per 100 m of flagging distance (unit: ticks per flag·100 m). To ensure adequate and comparable sampling effort, the total flagging distance within each sampling plot was no less than 500 m, and the sampling duration was no less than 30 minutes. Ticks attached to the cloth flag or the collector were removed using forceps and preserved in covered and labeled screw-top tubes. Tick specimens collected from the same sampling plot were pooled into the same tube or assigned consistent identification codes. The number of captured ticks were recorded in the field, and samples were transported to the laboratory for detailed species identification and classification based on morphological examination. Point-level observations were aggregated to county-month values by summing tick counts and corresponding sampling effort across plots within each county and month, and then calculating a standardized tick density index. Monthly tick abundance data from surveillance were used to establish the climate-abundance association. For years without surveillance data (2017–2018 and 2023), tick abundance was obtained from model-based estimates derived from the fitted model (see “Eco-epidemiological model”).

### SFTS cases

Case-level records of SFTS cases in Jiangsu Province from January 2017 to October 2023 were obtained from the Nationwide Notifiable Infectious Diseases Reporting Information System (NIDRIS). Cases were identified based on laboratory confirmation, including SFTSV RNA detection by PCR, seroconversion or a four-fold increase in antibody titers between paired serum samples collected at intervals of two weeks or more, and virus isolation from cell culture, or clinical diagnosis. Restricting to the laboratory-confirmed cases, a total of 782 cases were included in the analysis, of which 63 resulted in death. We collected information associated with each case, including the residential address, age, sex, occupation, date of illness onset and diagnosed, and history of tick exposure or bites. Tick exposure was defined based on epidemiological investigation records and referred to potential exposure within one month prior to illness onset, including visits to hilly or mountainous areas, engagement in agricultural activities in such environments, or the presence of ticks on domestic animals. Tick bite history specifically referred to self-reported tick bites. After data cleaning, no missing values remained for tick exposure or bite history in the analytical dataset. Case-level records were used for epidemiological analyses and identification of high-risk factors of case fatality (see “Epidemiological analyses” and “High-risk fatal factors analysis”). Using these records, we determined the aggregated number of cases per month per high-risk county for mathematical modelling analysis (see “Eco-epidemiological model”).

### Meteorological data

We acquired daily climate data including air temperature at 2 meter and total precipitation from the fifth-generation reanalysis (ERA5) provided by the European Center for Medium-Range Weather Forecasts (ECMWF) [16]. The meteorological data covered the period from 2017 to 2023, aligning with the temporal span of reported human SFTS cases. Time-series data were extracted by identifying the nearest grid point to the center of each local authority. This data was then aggregated to yield the monthly accumulated temperature and number of precipitation days for each county. The number of precipitation days was used to reflect the persistence of humid conditions relevant to tick survival and activity, rather than the intensity of rainfall events. These aggregations aided in deducing the climate-abundance association (see “Eco-epidemiological model”).

### Epidemiological analyses

We began by characterizing the evolving epidemic profile of SFTS human infections from 2017 to 2023. To do this, we first examined the spatial heterogeneities of infections, identifying endemic counties defined as those with reported SFTS cases in at least six years during 2017–2023 study period. Limiting subsequently analyses to these endemic counties, we further delineated disease spatial diffusions by quantifying the annual number of cases and infected towns. The changing severity of the infections was characterized by annual CFR over the study period. Additionally, we assessed the seasonality of human infections, taking into account potential variations in the seasonal profiles of epidemic trajectories over the observed period.

### Eco-epidemiological model

In line with the fundamental ecological dependence of vectored disease dynamics, we developed an eco-epidemiological model that connects natural environment, vector abundance and human cases. Specifically, we employed a statistical model to establish climate-abundance association; and sequentially developed a mechanistic epidemiological model to derive SFTS transmission among humans. The climate-abundance association was formed by incorporating local meteorological conditions and the long-term surveillance data of ticks into a generalized additive model (GAM) Eq (1). With the associations, we derived the monthly estimates of climate-based vectored abundance. We assumed that variations in vectored abundance is proportional to the seasonal transmission rate of SFTS in human populations. Therefore, vector abundance was used as a proxy for SFTS transmission rate in the seasonal susceptible–infected–recovered (SIR) model (Eqs (2)–(4)). The coupling model is described by the following equations:


$$\begin{array}{c} V_{t,i}=a+b({lon}_{i},{lat}_{i})+c(T_{t-1,i})+d(P_{t-1,i})+\varepsilon_{t,i} \end{array}$$ (1)

$$\begin{array}{c} \frac{dS}{dt}=-\frac{\beta^{\prime }(t)\hat{V}SI}{N} \end{array}$$ (2)

$$\begin{array}{c} \frac{dI}{dt}=\frac{\beta^{\prime }(t)\hat{V}SI}{N}-\gamma I \end{array}$$ (3)

$$\begin{array}{c} \frac{dR}{dt}=\gamma I \end{array}$$ (4)

where $V_{t}$ is the vector abundance in month t in county i. $b\left({lon}_{i},{lat}_{i}\right)$ is a two-dimensional component characterizing spatial heterogeneity. Given the roughly 5-week growth and development period of ticks [17], the model includes a 1-month lag between the local climate conditions and vector population dynamics. Such that $c(T_{t-1,i})$ and $d(P_{t-1,i})$ captures the monthly accumulated temperature and number of rainy days in the previous month, respectively. The parameters a and $\varepsilon_{t,i}$ denote the overall intercept and model error, respectively. Model diagnostics indicated an adequate fit of the GAM, with approximately 36% deviance explained and no evidence of overfitting based on estimated degrees of freedom. A quasi-Poisson specification was used to account for overdispersion. Moreover, mechanistic model is consisted of three components, i.e., the susceptible (S), infected (I), and recovered (R) individuals in human populations. Thus, the population size is calculated as N=S+I+R. $\hat{V}$ represents the estimates of tick abundance using Eq (1) and is used as a proxy to introduce seasonal and spatial variation in transmission intensity in the SIR model. Transmission rate among humans is collectively determined by $\beta^{\prime }(t)$ and $\hat{V}$ using ${\beta (t)=\beta }^{\prime }(t)\hat{V}$. In this case, $\beta^{\prime }(t)$ represents a time-varying transmission efficiency. It is modelled as a scaling factor that links estimated tick abundance to the transmission rate in the SIR model and captures multiple unresolved influences on human transmission, such as human behavior, tick infection prevalence, or habitat factors. The average infectious period ($\frac{1}{\gamma }$) is modelled as a random constant varying from 10–20 days [3,18]. Consequently, the effective reproductive ratio was calculated as $R_{e}=\frac{{(R}_{0}S(t))}{N}$, where $R_{0}=\frac{\beta^{\prime }(t)\hat{V}}{\gamma }$.

We calibrated the GAM using monthly tick abundance and mean climatic data from 2017 to 2023. We additionally calibrated the SIR model to the recorded cases during significant outbreak years (defined as years where reported cases exceeded the county-specific median during the study period) in endemic counties. We assume homogeneous susceptibility within human populations, justified by the low background immunity in the general population, and reinitialised the model 500 times with varying γ values at the beginning of each outbreak year. The median estimates and 95% confidence intervals of cases from all simulations using varying γ values are presented. Additionally, we estimated the annual average magnitudes of $R_{e}$ to assess the county-specific transmission potential. County-specific median estimates of vector efficacy were derived, and then rescaled as relative vector efficacy as compared with the overall efficacy using ${RVE}_{c}=\frac{{VE}_{c}}{\overset{―}{{VE}_{c}}}$, where ${VE}_{c}$ is vector efficacy in county c and $\overset{―}{{VE}_{c}}$ is average vector efficacy among counties, years and infectious period, ensuing the relative vector efficacy fluctuates around a mean of 1.

### High-risk fatal factors analysis

We incorporated multifaceted factors in the analysis, including demographic characteristics (i.e., age, sex and occupation), tick abundance, tick exposure history, and the duration between onset and diagnosis. To systematically select variables associated with fatal outcomes after infection, the least absolute shrinkage and selection operator (LASSO) was first applied for variable selection using the *glmnet* package in R. The full set of candidate variables initially considered included age group (<65 years, ≥ 65 years), gender (male, female), occupation (farmer, other), duration from symptom onset to diagnosis (<7 days, ≥ 7 days), and tick exposure history (yes, no), and neighborhood tick abundance in the current and previous month (as a continuous variable). All continuous variables were standardized prior to model fitting. The optimal penalization parameter (λ) was selected using 10-fold cross-validation, with the value minimizing the cross-validated deviance (λ = 0.009) chosen for the final model. Variables retained after LASSO selection were subsequently entered into a multivariable logistic regression to quantify their associations with the odds of case fatality after infection.

## Results

The Results are structured to follow the analytical workflow described in the Methods, beginning with descriptive epidemiological analyses, followed by modelling of the association between climate variables and estimated tick abundance, eco-epidemiological transmission modelling, and finally case-level analysis of case fatality risk factors.

### Study population

A total of 782 laboratory-confirmed human infections were included in our study, including 389 male and 393 female patients. The overall age profile of the infections shows that the median age of the documented patients is 66 years old. Importantly, people aged 60–69 years associates with a higher incidence (S1 Table).

### Epidemiological dynamics of SFTS

**Fig 1:**
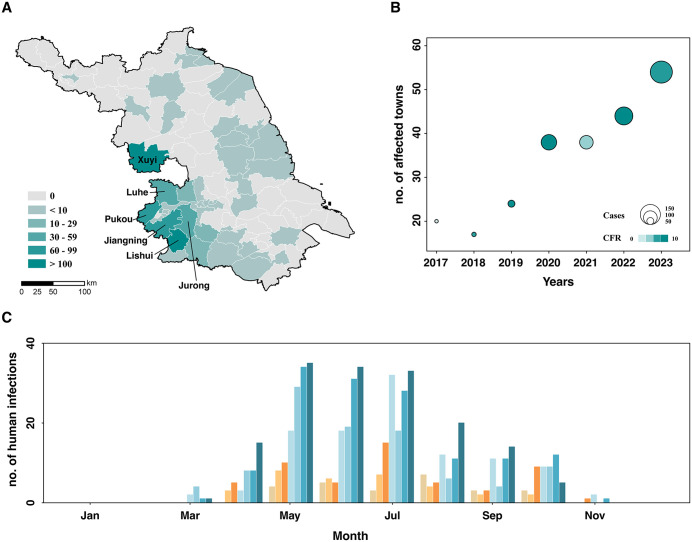
Epidemiological dynamics of SFTS in Jiangsu in 2017-2023. **(A)** County-level number of laboratory-confirmed SFTS human cases presents the spatial heterogeneity of disease risks (https://cloudcenter.tianditu.gov.cn/administrativeDivision/, Map Review No. GS (2024) 0650). **(B)** Among endemic counties, epidemiological dynamics of the disease is characterized by (B) the annual number of human cases, CFR and affected towns; as well as the **(C)** seasonality.

Surveillance of human SFTS infections reveals distinct spatial heterogeneity across counties (Fig 1A). Precisely, we observed a typically larger number of human infections in six counties: Xuyi, Lishui, Jiangning, Pukou, Luhe and Jurong, which collectively account for approximately 80% of the total infections (S2 Table). Additionally, these endemic counties are characterized by a longer persistence of infections than others, exhibiting over six years of continuous disease circulation among humans (S2 Table). Focusing on these endemic counties, we show a substantial increase in the annual number of cases, rising by 572% from 25 cases in 2017–168 cases in 2023, alongside an increase in the number of infected towns (Fig 1B). Importantly, this uptick coincides with a growing severity of the disease (Fig 1B), with the annual CFR increasing from 0% to roughly 10% over the past years. Furthermore, the dynamics of SFTS exhibit strong seasonality within endemic counties (Fig 1C). It is also worth noting that such seasonality varies, yielding shifts in the peak occurrence from July to May since 2021.

### Climate-based vectored dynamic risks

**Fig 2:**
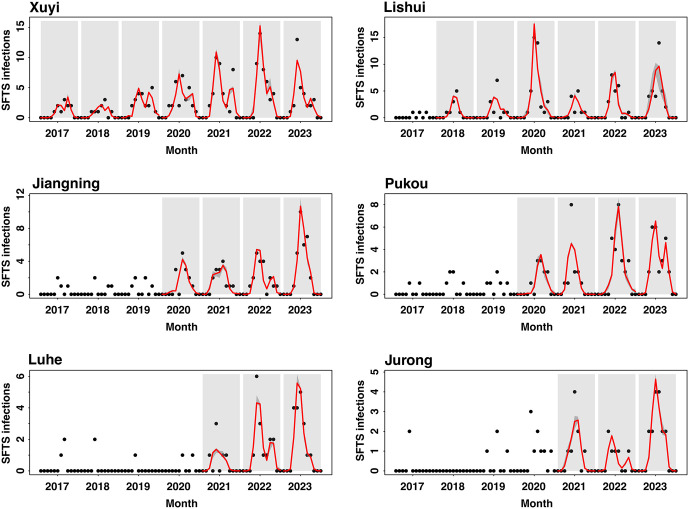
Model inference of SFTS human cases among endemic counties. The observed number of laboratory-confirmed human cases (black points) in outbreak years (the grey shaded area) is used for model simulation. The model was reinitialized by using a plausible range of infectious periods at the beginning of each outbreak year. The median estimates of human cases (red lines) and corresponding confidence intervals are presented.

By incorporating climate-based vectored transmission rate in our seasonal epidemiological model, we operationalize a quantitative association between estimated vector abundance and SFTS transmission. The GAM revealed a significant non-linear association between accumulated temperature (lagged by one month) and estimated tick abundance, whereas the effect of precipitation days (lagged by one month) was weaker and not statistically significant (S1 Fig). Overall, the model reproduced the main temporal patterns of observed disease dynamics across years and counties (Figs 2 and S2). Therefore, our model adeptly portrays the seasonal dynamics of human SFTS across diverse climatic and ecological contexts.

**Fig 3:**
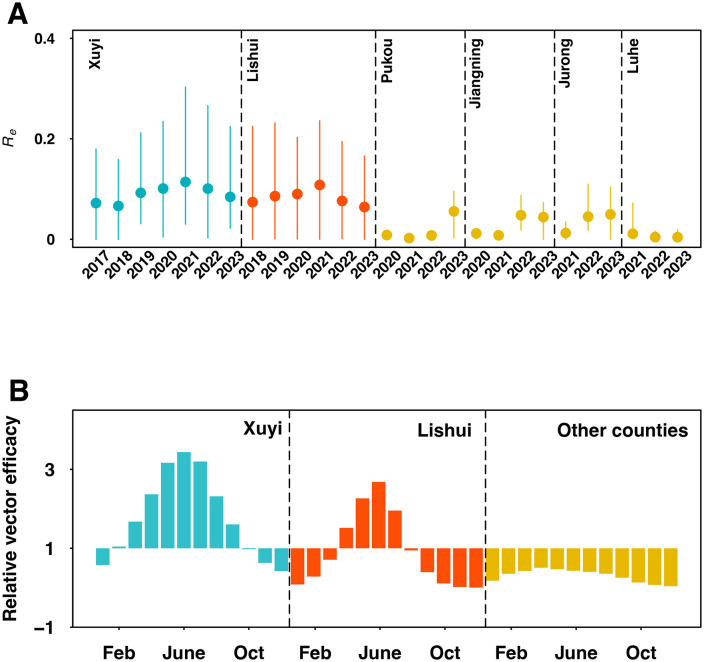
Estimates of the transmission potential of SFTS among endemic counties. **(A)** The median estimates and confidence intervals of $R_{e}$ in outbreak years capture the overall transmission potential of SFTS among humans. **(B)** Monthly estimates of vector efficacy characterize the relative capacity across counties.

Further investigation indicates heterogeneity in the transmission potential of SFTS among endemic counties. For instance, the median estimates of $R_{e}$ in Xuyi and Lishui, depending on outbreak years, is 0.10 (95% CI: 0.001–0.224). This contrasts with a median of $R_{e}$ < 0.01 in the other four counties (Figs 3A and S3A). Consistent with these disparities, our estimates shows that the median estimates of the peaking magnitude of $R_{e}$ over the years is around 0.3 in Xuyi and Lishui, while it remains below 0.1 in other counties (S3B Fig).

It is noteworthy that these spatial differences in $R_{e}$ coincide with the higher vector efficacy in Xuyi and Lishui. As compared with the overall transmission efficacy spanning various years and counties, there is evidence of a notably higher efficacy from March to September in these two counties (Fig 3B). Given the relatively limited variation in estimated vector abundance across counties (S4 Fig), the higher efficacy and $R_{e}$ in Xuyi and Lishui suggest additional heterogeneity in transmission intensity beyond that captured by modeled abundance alone. Investigating the tick positivity and exposure history, unveils higher tick positivity and a larger fraction of patients with tick exposure or biting history in Xuyi and Lishui (S5 Fig). This implies a possible collective contribution to the heightened vector efficacy observed.

### Multifaceted determinants of case fatality after infection

After adjusting for occupation, onset-diagnosis duration and tick abundance, older age correlates with significantly increased odds of death after infection (Table 1). Our estimates show that people over 65 years would have 197.9% (43.3%–582.5%) higher odds of death than those under 65 years. Notably, patients living in the neighbourhood with higher estimated tick abundance showed higher odds of deaths after infection. It is estimated that higher level of tick abundance is associated with approximately 202.8% higher odds of death. Comparatively, we found no evidence of a significant effect from occupation and onset-diagnosis duration on the odds of case fatality.

**Table 1:** Potential factors associated with the risk of fatal outcomes. Three sets of factors were included: demographic factors (age, sex, occupation), tick abundance in the neighbourhood environment, care-seeking behaviour (or the duration from onset to diagnosis). Median estimates and corresponding 95% CIs of each factor are provided. Numbers with “*” indicate statistically significant (p < 0.05).

Factors	OR (95%CI)
Age, years	
<65	Reference group
≥65	2.979* (1.433, 6.825)
Occupation	
Others	Reference group
Farmers	0.906 (0.445, 1.970)
Duration from onset to diagnosis, days	
<7	Reference group
≥7	0.922 (0.478, 1.775)
Tick abundance	3.028* (1.625, 5.741)

## Discussion

Our study aligns with the observed increase in SFTS cases in most recent years [14,15]. Crucially, we observed shifts in the epidemic profile of the disease, with a substantial increase in human infections, the number of affected areas, and disease severity. These findings are broadly consistent with other studies done across a variety of settings [19–21]. Importantly, the seasonality of the disease is not static, and can shift over time. While the incidence of human cases might typically be higher from May to August, there has been a notable peak around May starting in 2021. This shift may be associated with earlier seasonal tick activity and changes in human outdoor behaviour in recent years, including altered activity patterns following the COVID-19 pandemic, which could advance the timing of human exposure to ticks [15]. Therefore, we provide the first empirical evidence of the evolving epidemiological dynamics of SFTS. These insights emphasize the need to investigate the potential changes in future disease dynamics among broader ecological settings.

A primary challenge in assessing SFTS risk involves contextualizing epidemiological dynamics within local ecological context. The novelty of our study lies in its inherently multidisciplinary nature and cohesiveness, harnessing a suite of evidence from disparate perspectives across the fields of ecology and epidemiology. By integrating extensive data streams to eco-epidemiological model, we demonstrate how local weather conditions, through associations with estimated tick abundance, may contribute to observed variations in SFTS dynamics in China (see S1 Text). While these climate–abundance associations highlight the role of meteorological conditions in shaping seasonal variation in tick abundance, they represent only the component of variability that can be explained by climate. Tick abundance is influenced by a broader set of interacting biotic and abiotic factors, including host availability, habitat structure, and land-use patterns, which were not explicitly modelled in this study. This framing first allows us to gain insight of vector population dynamics in response to local weather condition; it then aids in assessing the non-linear effects of vector abundance on SFTS dynamics. Therefore, our predictive framework offers a route to placing tick-borne disease dynamics in an ecological system.

Although the estimated $R_{e}$ values are consistently below one, this does not contradict the observed persistence of SFTS cases across multiple years. Humans are considered dead-end hosts for SFTSV, and sustained transmission within the human population is not expected. Instead, recurrent seasonal spillover from infected ticks can generate observable outbreaks each year, even when the effective reproduction number remains below one. In this context, $R_{e}$ should be interpreted as a relative indicator of transmission potential shaped by ecological and seasonal forcing, rather than a threshold parameter for epidemic persistence. While $R_{e}$ provides a useful relative indicator of transmission potential within counties over time, comparisons of absolute $R_{e}$ values across counties should be interpreted cautiously due to differences in ecological context, surveillance intensity, and reporting.

It is notable that vector efficacy and thus transmission potential is typically higher in Xuyi and Lishui compared to other counties, coinciding with higher tick positivity and increased likelihood of human exposure. Recent years have witnessed crucial developments in understanding of how the spatiotemporal dynamics of SFTS depend on a multitude of interacting ecological factors. It has become evident that mountainous region, especially at the border of Jiangsu and Anhui provinces, pose a heightened risk of SFTS infections [15,22]. However, such models tend to rely heavily on the statistical interplay with ecological factors without fully encapsulating the inherent epidemiological processes. We argue that opportunities exist to more deliberately adopt an eco-epidemiological approach in SFTS mechanistic transmission and risk assessment. The success of our model inference, combined with large-scale vector surveillance and lab experiment, opens up the possibility of applying eco-epidemiological approach to the real-time risk assessment of zoonotic diseases.

Notably, the key to improving public health decisions is to recognize the determinants of the epidemiological trajectories of the disease. Most of the existing models have tended to improve risk assessment by relying on multiple parameters [23]. However, the complexity of such models may inevitably yield biases when identifying key determinants. To address this, we specifically modelled the rate of transmission using vector efficacy and abundance and linked the epidemiological interpretation of the efficacy to tick positivity and human exposure history. Within this framework, vector efficacy should be understood as a composite, population-level indicator that reflects transmission efficiency under local ecological and behavioural contexts, rather than a direct biological property of ticks or a causal determinant at the individual level. Our model simulations highlight that tick positivity and human exposure are tightly tied to vector efficacy and thus seasonal transmission potential of SFTS. Attention to this joint signature of ticks and human behavioural risk factors are especially relevant to designing targeted strategies for SFTS risk management. Existing evidence have suggested that preventive measures should be primarily directed towards to people exhibiting high-risk behaviours, such as those with occupational exposure and those engaging outdoor activities [24–26].

Beyond ecological observations, our findings offer crucial insights for clinical studies. Historically, clinical scoring systems have contributed to the prediction of fatal outcome [27–29]. Of the factors included in such scoring system, age of patients has been recognized as the key to determining their vulnerability to death. Consistent with this, we identified that patients over 65 years old associate with higher risk of fatal outcomes. This means that senior patients should be prioritized for diagnosis and treatment in clinical settings. It is worth noting that our findings not only support the well-recognized signature of age on the risk fatal outcomes, but also propose the significance of considering tick abundance in the neighbourhood of residence in clinical activities. We show a considerable excess risk for patients living in the neighbourhood of higher tick abundance. Therefore, tick abundance in the residential area may provide contextual information for population level risk assessment and targeted prevention strategies, rather than serving as a direct clinical predictor. Tick abundance was estimated from a climate-informed statistical model rather than directly observed, and the associated uncertainty is implicitly carried forward into subsequent analyses. However, uncertainty from the statistical model was not explicitly propagated into the fatality model, and measurement error in estimated abundance may attenuate the observed association with fatal outcomes. Accordingly, associations involving tick abundance and vector efficacy should be interpreted cautiously as population-level, non-causal relationships reflecting broader ecological and potentially socio-demographic contexts, such as rurality, healthcare access, or unmeasured comorbidities.

Our study highlights opportunities for future development. We demonstrate essential role of vector efficacy in transmission. However, some ecological studies suggest vector population expansion would be the key in other settings [30]. Thus, one focus for future research would be assessing the relative role of vector efficacy and abundance in diverse settings. In addition, while the present analysis focused on overall tick abundance, future studies incorporating species-specific tick surveillance data may provide further ecological and mechanistic insight, and help refine the interpretation of transmission heterogeneity in eco-epidemiological modelling. Explicitly incorporating tick positivity into mechanistic transmission model may have the potential to implicating the interacting processes across scales and risk assessment. Furthermore, the increase in SFTS incidence and case fatality over the most recent years might partially attributable to the improved health system, changing care-seeking behavior, and the increased outdoor activities. It is crucial to evaluate their alterations on disease epidemiology.

The key messages of this study are of great public health implications. We make critical contributions to understanding how epidemiological profile may have evolved over time. Crucially, this study helps reveal how ecological contexts has disproportionately affected SFTS epidemiological dynamics across empirical settings. This represents a promising juncture between disease ecology and epidemiology. ‌‌Such evidence can guide health authorities to grasp the intricacies of diverse epidemic risks, and ultimately facilitating the prioritization of resources to the most at-risk regions and populations.

## Supporting information

S1 Fig Partial effects of climatic factors on estimated tick abundance. Non-linear associations between lagged accumulated temperature (A) and lagged precipitation days (B) with estimated tick abundance derived from the generalized additive model. Dashed lines indicate 95% confidence intervals.
(DOCX)

S2 Fig The accuracy of the inference of SFTS human infections in 2017–2023. The number of the observed and estimated human infections in each endemic counties and all endemic counties is presented. The accuracy of the inference is assessed using RMSE.
(DOCX)

S3 FigSpatial disparities in the estimates of $R_{e}$.(A) The monthly estimates and (B) the Peaking magnitude of $R_{e}$ in large outbreak years in 2017–2023 are distinguished among six endemic counties.(DOCX)

S4 Fig Dynamics of tick abundance across endemic counties and years. County-specific median estimates of tick abundance over years were rescaled as relative abundance as compared with the overall abundance over years in each county.
(DOCX)

S5 Fig Signature of ticks and human behavioural risk factors on SFTS human infections. (A) The positivity of ticks and (B) the fraction of humans with exposure or biting history is evaluated by lab experiments and survey. Estimates in Lishui and Xuyi are compared with those in other four endemic counties (i.e., Pukou, Jiangning, Jurong and Luhe).
(DOCX)

S1 Table Demographic and age distribution of SFTS cases (n = 782). (DOCX)

S2 Table Spatial distribution of SFTS human infections in 2017–2023. The proportion of infections in six endemic counties is presented. The number of years with recorded human infections shows the persistence of disease circulation in the study period.
(DOCX)

S3 Table Model fitting of the tick abundance. The performance of statistical model with one-month and two-month lag between local meteorological conditions and tick abundance is quantified by the generalized cross-validation criterion (GCV), the proportion of deviation explained by model and the significant weather predictors (p < 0.05).
(DOCX)

S4 Table Model fitting of human infections. The number of human infections in each endemic counties and all endemic counties is estimated using two mechanistic models, i.e., the climate-based vectored transmission model and “vector-free” model. The accuracy of the inference is assessed by comparing the observed and the estimated number of human infections using RMSE.
(DOCX)

S1 Text Sensitivity analyses. (DOCX)

## References

[1] Yu X-J, Liang M-F, Zhang S-Y, Liu Y, Li J-D, Sun Y-L, et al. Fever with thrombocytopenia associated with a novel bunyavirus in China. N Engl J Med. 2011;364(16):1523–32. doi: 10.1056/NEJMoa1010095 21410387 PMC3113718

[2] Xu B, Liu L, Huang X, Ma H, Zhang Y, Du Y, et al. Metagenomic analysis of fever, thrombocytopenia and leukopenia syndrome (FTLS) in Henan Province, China: discovery of a new bunyavirus. PLoS Pathog. 2011;7(11):e1002369. doi: 10.1371/journal.ppat.1002369 22114553 PMC3219706

[3] Li J-C, Zhao J, Li H, Fang L-Q, Liu W. Epidemiology, clinical characteristics, and treatment of severe fever with thrombocytopenia syndrome. Infect Med (Beijing). 2022;1(1):40–9. doi: 10.1016/j.imj.2021.10.001 38074982 PMC10699716

[4] World Health Organization. 2017 Annual Review of Diseases Prioritized Under the Research and Development Blueprint. 2017.

[5] Li R, Xu L, Bjørnstad ON, Liu K, Song T, Chen A, et al. Climate-driven variation in mosquito density predicts the spatiotemporal dynamics of dengue. Proc Natl Acad Sci U S A. 2019;116(9):3624–9. doi: 10.1073/pnas.1806094116 30808752 PMC6397594

[6] Li R, Su C, Lou Z. Associations between ecological diversity and rodent plague circulation in Yunnan Province, China, 1983–2020: A data-informed modelling study. PLoS Negl Trop Dis. 2023;17:e0011317–e0011317. 10.1371/journal.pntd.0011317 PMC10287002 37347759

[7] Li D. A highly pathogenic new bunyavirus emerged in China. Emerg Microbes Infect. 2013;2(1):e1. doi: 10.1038/emi.2013.1 26038435 PMC3630492

[8] Zhang X, Zhao C, Cheng C, Zhang G, Yu T, Lawrence K, et al. Rapid spread of severe fever with thrombocytopenia syndrome virus by parthenogenetic Asian Longhorned Ticks. Emerg Infect Dis. 2022;28(2):363–72. doi: 10.3201/eid2802.211532 35075994 PMC8798674

[9] Ding F-Y, Ge H-H, Ma T, Wang Q, Hao M-M, Li H, et al. Projecting spatiotemporal dynamics of severe fever with thrombocytopenia syndrome in the mainland of China. Glob Chang Biol. 2023;29(23):6647–60. doi: 10.1111/gcb.16969 37846616

[10] Miao D, Liu M-J, Wang Y-X, Ren X, Lu Q-B, Zhao G-P, et al. Epidemiology and ecology of severe fever with thrombocytopenia syndrome in China, 2010‒2018. Clin Infect Dis. 2021;73(11):e3851–8. doi: 10.1093/cid/ciaa1561 33068430 PMC8664468

[11] Liu W, Dai K, Wang T, Zhang H, Wu J, Liu W, et al. Severe fever with thrombocytopenia syndrome incidence could be associated with ecotone between forest and cultivated land in rural settings of central China. Ticks Tick Borne Dis. 2023;14(2):102085. doi: 10.1016/j.ttbdis.2022.102085 36435169

[12] Liu K, Cui N, Fang L-Q, Wang B-J, Lu Q-B, Peng W, et al. Epidemiologic features and environmental risk factors of severe fever with thrombocytopenia syndrome, Xinyang, China. PLoS Negl Trop Dis. 2014;8(5):e2820. doi: 10.1371/journal.pntd.0002820 24810269 PMC4014392

[13] Zhang D, Sun C, Yu H, Li J, Liu W, Li Z, et al. Environmental risk factors and geographic distribution of severe fever with thrombocytopenia syndrome in Jiangsu Province, China. Vector Borne Zoonotic Dis. 2019;19(10):758–66. doi: 10.1089/vbz.2018.2425 30994412

[14] Liang S, Xie W, Li Z. Analysis of fatal cases of severe fever with thrombocytopenia syndrome in Jiangsu province, China, between 2011 and 2022: a retrospective study. Front Public Health. 2023;11. 10.3389/fpubh.2023.1076226 PMC10076888 37033043

[15] Liang S, Li Z, Zhang N, et al. Epidemiological and spatiotemporal analysis of severe fever with thrombocytopenia syndrome in Eastern China, 2011–2021. BMC Public Health 2023;23. 10.1186/s12889-023-15379-3 PMC10019416 36927782

[16] Hersbach H, Bell B, Berrisford P, et al. The ERA5 global reanalysis. Q J Roy Meteor Soc. 2020;146.

[17] Meng H, Xu S, Yu Z, Liu Z, Liu J, Yang X, et al. The life cycle and occurrence of Haemaphysalis concinna (Acari: Ixodidae) under field conditions. Ticks Tick Borne Dis. 2014;5(6):887–91. doi: 10.1016/j.ttbdis.2014.07.007 25113978

[18] Kwon J-S, Kim M-C, Kim JY, Jeon N-Y, Ryu B-H, Hong J, et al. Kinetics of viral load and cytokines in severe fever with thrombocytopenia syndrome. J Clin Virol. 2018;101:57–62. doi: 10.1016/j.jcv.2018.01.017 29427908 PMC7106421

[19] Li H, Lu Q-B, Xing B, Zhang S-F, Liu K, Du J, et al. Epidemiological and clinical features of laboratory-diagnosed severe fever with thrombocytopenia syndrome in China, 2011-17: a prospective observational study. Lancet Infect Dis. 2018;18(10):1127–37. doi: 10.1016/S1473-3099(18)30293-7 30054190

[20] Kim J, Hong H-J, Hwang J-H, Shin N-R, Hwang K. Risk factors associated with death due to severe fever with thrombocytopenia syndrome in hospitalized Korean patients (2018-2022). Osong Public Health Res Perspect. 2023;14(3):151–63. doi: 10.24171/j.phrp.2023.0048 37415432 PMC10522827

[21] Huang X, Li J, Li A, Wang S, Li D. Epidemiological characteristics of severe fever with thrombocytopenia syndrome from 2010 to 2019 in Mainland China. Int J Environ Res Public Health. 2021;18(6):3092. doi: 10.3390/ijerph18063092 33802869 PMC8002760

[22] You E, Wang L, Zhang L, Wu J, Zhao K, Huang F. Epidemiological characteristics of severe fever with thrombocytopenia syndrome in Hefei of Anhui Province: a population-based surveillance study from 2011 to 2018. Eur J Clin Microbiol Infect Dis. 2021;40(5):929–39. doi: 10.1007/s10096-020-04098-x 33188497

[23] Zhang N, Cheng X-Q, Deng B, Rui J, Qiu L, Zhao Z, et al. Modelling the transmission dynamics of severe fever with thrombocytopenia syndrome in Jiangsu Province, China. Parasit Vectors. 2021;14(1):237. doi: 10.1186/s13071-021-04732-3 33957950 PMC8100741

[24] Xiong W-Y, Feng Z-J, Matsui T, Foxwell AR. Risk assessment of human infection with a novel bunyavirus in China. Western Pac Surveill Response J. 2012;3(4):61–6. doi: 10.5365/WPSAR.2012.3.4.002 23908943 PMC3729083

[25] Shin J, Kwon D, Youn S-K, Park J-H. Characteristics and factors associated with death among patients hospitalized for severe fever with thrombocytopenia syndrome, South Korea, 2013. Emerg Infect Dis. 2015;21(10):1704–10. doi: 10.3201/eid2110.141928 26402575 PMC4593431

[26] Hu J-L, Li Z-F, Wang X-C, Hong L, He H, Chen W-G, et al. Risk factors for bunyavirus-associated severe fever with thrombocytopenia syndrome: a community-based case-control study. PLoS One. 2016;11(11):e0166611. doi: 10.1371/journal.pone.0166611 27846273 PMC5112944

[27] Jia B, Yan X, Chen Y, Wang G, Liu Y, Xu B, et al. A scoring model for predicting prognosis of patients with severe fever with thrombocytopenia syndrome. PLoS Negl Trop Dis. 2017;11(9):e0005909. doi: 10.1371/journal.pntd.0005909 28934195 PMC5626493

[28] Song P, Zheng N, Liu Y, Tian C, Wu X, Ma X, et al. Deficient humoral responses and disrupted B-cell immunity are associated with fatal SFTSV infection. Nat Commun. 2018;9(1):3328. doi: 10.1038/s41467-018-05746-9 30127439 PMC6102208

[29] Gai Z-T, Zhang Y, Liang M-F, Jin C, Zhang S, Zhu C-B, et al. Clinical progress and risk factors for death in severe fever with thrombocytopenia syndrome patients. J Infect Dis. 2012;206(7):1095–102. doi: 10.1093/infdis/jis472 22850122

[30] Deng B, Rui J, Liang S-Y, Li Z-F, Li K, Lin S, et al. Meteorological factors and tick density affect the dynamics of SFTS in jiangsu province, China. PLoS Negl Trop Dis. 2022;16(5):e0010432. doi: 10.1371/journal.pntd.0010432 35533208 PMC9119627

