# Supplementary material for: Ecological signature on the epidemiological dynamics of severe fever with thrombocytopenia syndrome
Source: PLoS Negl Trop Dis. 2026 Jun 8;20(6):e0014408. doi: 10.1371/journal.pntd.0014408 (PMC13245741; doi:10.1371/journal.pntd.0014408)
Supplement: S1 Fig — Non-linear associations between lagged accumulated temperature (A) and lagged precipitation days (B) with estimated tick abundance derived from the generalized additive model. Dashed lines indicate 95% confidence intervals. (DOCX) [file pntd.0014408.s001.docx]

**S1 Fig. Partial effects of climatic factors on estimated tick abundance.** Non-linear associations between lagged accumulated temperature (A) and lagged precipitation days (B) with estimated tick abundance derived from the generalized additive model. Dashed lines indicate 95% confidence intervals.
