# Supplementary material for: Ecological signature on the epidemiological dynamics of severe fever with thrombocytopenia syndrome
Source: PLoS Negl Trop Dis. 2026 Jun 8;20(6):e0014408. doi: 10.1371/journal.pntd.0014408 (PMC13245741; doi:10.1371/journal.pntd.0014408)
Supplement: S2 Fig — The number of the observed and estimated human infections in each endemic counties and all endemic counties is presented. The accuracy of the inference is assessed using RMSE. (DOCX) [file pntd.0014408.s002.docx]

**S2 Fig. The accuracy of the inference of SFTS human infections in 2017-2023.** The number of the observed and estimated human infections in each endemic counties and all endemic counties is presented. The accuracy of the inference is assessed using RMSE.
