# Supplementary material for: Ecological signature on the epidemiological dynamics of severe fever with thrombocytopenia syndrome
Source: PLoS Negl Trop Dis. 2026 Jun 8;20(6):e0014408. doi: 10.1371/journal.pntd.0014408 (PMC13245741; doi:10.1371/journal.pntd.0014408)
Supplement: S3 Fig — (A) The monthly estimates and (B) the Peaking magnitude of Re in large outbreak years in 2017–2023 are distinguished among six endemic counties. (DOCX) [file pntd.0014408.s003.docx]

**S3 Fig. Spatial disparities in the estimates of** $\boldsymbol{R}_{\boldsymbol{e}}$**.** (A) The monthly estimates and (B) the Peaking magnitude of $R_{e}$ in large outbreak years in 2017-2023 are distinguished among six endemic counties.
