# Supplementary material for: Ecological signature on the epidemiological dynamics of severe fever with thrombocytopenia syndrome
Source: PLoS Negl Trop Dis. 2026 Jun 8;20(6):e0014408. doi: 10.1371/journal.pntd.0014408 (PMC13245741; doi:10.1371/journal.pntd.0014408)
Supplement: S4 Fig — County-specific median estimates of tick abundance over years were rescaled as relative abundance as compared with the overall abundance over years in each county. (DOCX) [file pntd.0014408.s004.docx]

**S4 Fig. Dynamics of tick abundance across endemic counties and years.** County-specific median estimates of tick abundance over years were rescaled as relative abundance as compared with the overall abundance over years in each county.
