# Supplementary material for: Ecological signature on the epidemiological dynamics of severe fever with thrombocytopenia syndrome
Source: PLoS Negl Trop Dis. 2026 Jun 8;20(6):e0014408. doi: 10.1371/journal.pntd.0014408 (PMC13245741; doi:10.1371/journal.pntd.0014408)
Supplement: S5 Fig — (A) The positivity of ticks and (B) the fraction of humans with exposure or biting history is evaluated by lab experiments and survey. Estimates in Lishui and Xuyi are compared with those in other four endemic counties (i.e., Pukou, Jiangning, Jurong and Luhe). (DOCX) [file pntd.0014408.s005.docx]

**S5 Fig. Signature of ticks and human behavioural risk factors on SFTS human infections.** (A) The positivity of ticks and (B) the fraction of humans with exposure or biting history is evaluated by lab experiments and survey. Estimates in Lishui and Xuyi are compared with those in other four endemic counties (i.e. Pukou, Jiangning, Jurong and Luhe).
