# Supplementary material for: Ecological signature on the epidemiological dynamics of severe fever with thrombocytopenia syndrome
Source: PLoS Negl Trop Dis. 2026 Jun 8;20(6):e0014408. doi: 10.1371/journal.pntd.0014408 (PMC13245741; doi:10.1371/journal.pntd.0014408)
Supplement: S1 Table — (DOCX) [file pntd.0014408.s006.docx]

**S1 Table. Demographic and age distribution of SFTS cases (n = 782).**

|  | **All cases**  **(n = 782)** | **Male**  **(n = 389)** | **Female**  **(n = 393)** |
| --- | --- | --- | --- |
| **Median age** | 66 | 66 | 67 |
| **Age group, years** |  |  |  |
| 0-9 | 3 | 2 | 1 |
| 10-19 | 1 | 0 | 1 |
| 20-29 | 6 | 4 | 2 |
| 30-39 | 19 | 14 | 5 |
| 40-49 | 46 | 26 | 20 |
| 50-59 | 185 | 89 | 96 |
| 60-69 | 244 | 127 | 117 |
| 70-79 | 239 | 113 | 126 |
| 80-89 | 38 | 14 | 24 |
| Over 90 | 1 | 0 | 1 |
