# Supplementary material for: Ecological signature on the epidemiological dynamics of severe fever with thrombocytopenia syndrome
Source: PLoS Negl Trop Dis. 2026 Jun 8;20(6):e0014408. doi: 10.1371/journal.pntd.0014408 (PMC13245741; doi:10.1371/journal.pntd.0014408)
Supplement: S2 Table — The proportion of infections in six endemic counties is presented. The number of years with recorded human infections shows the persistence of disease circulation in the study period. (DOCX) [file pntd.0014408.s007.docx]

**S2 Table. Spatial distribution of SFTS human infections in 2017-2023.** The proportion of infections in six endemic counties is presented. The number of years with recorded human infections shows the persistence of disease circulation in the study period.

| Counties | Human infections | | Persistence (year) |
| --- | --- | --- | --- |
|  | Proportion (%) | Cumulative proportion (%) |  |
| Xuyi | 23.91 | 23.91 | 7 |
| Lishui | 17.90 | 41.82 | 7 |
| Jiangning | 12.02 | 53.84 | 7 |
| Pukou | 11.76 | 65.60 | 7 |
| Luhe | 6.52 | 72.12 | 7 |
| Jurong | 6.39 | 78.52 | 6 |
