# Supplementary material for: Ecological signature on the epidemiological dynamics of severe fever with thrombocytopenia syndrome
Source: PLoS Negl Trop Dis. 2026 Jun 8;20(6):e0014408. doi: 10.1371/journal.pntd.0014408 (PMC13245741; doi:10.1371/journal.pntd.0014408)
Supplement: S3 Table — The performance of statistical model with one-month and two-month lag between local meteorological conditions and tick abundance is quantified by the generalized cross-validation criterion (GCV), the proportion of deviation explained by model and the significant weather predictors (p < 0.05). (DOCX) [file pntd.0014408.s008.docx]

**S3 Table. Model fitting of the tick abundance.** The performance of statistical model with one-month and two-month lag between local meteorological conditions and tick abundance is quantified by the generalized cross-validation criterion (GCV), the proportion of deviation explained by model and the significant weather predictors (p < 0.05).

| Model | Model formula | GCV | Deviance explained (%) |
| --- | --- | --- | --- |
| One-month lag | $V_{t,i}=a_{t,i}+b({lon}_{i},{lat}_{i})+c(T_{t-1,i})+d(P_{t-1,i})+\varepsilon_{t,i}$ | 39.063 | 36.3% |
| Two-month lag | $V_{t,i}=a_{t,i}+b({lon}_{i},{lat}_{i})+c(T_{t-2,i})+d(P_{t-2,i})+\varepsilon_{t,i}$ | 45.003 | 23.2% |
