# Supplementary material for: Ecological signature on the epidemiological dynamics of severe fever with thrombocytopenia syndrome
Source: PLoS Negl Trop Dis. 2026 Jun 8;20(6):e0014408. doi: 10.1371/journal.pntd.0014408 (PMC13245741; doi:10.1371/journal.pntd.0014408)
Supplement: S4 Table — The number of human infections in each endemic counties and all endemic counties is estimated using two mechanistic models, i.e., the climate-based vectored transmission model and “vector-free” model. The accuracy of the inference is assessed by comparing the observed and the estimated number of human infections using RMSE. (DOCX) [file pntd.0014408.s009.docx]

**S4 Table. Model fitting of human infections.** The number of human infections in each endemic counties and all endemic counties is estimated using two mechanistic models, i.e. the climate-based vectored transmission model and “vector-free” model. The accuracy of the inference is assessed by comparing the observed and the estimated number of human infections using RMSE.

| Counties | RMSE | |
| --- | --- | --- |
|  | climate-based vectored transmission model | “vector-free” model |
| Xuyi | 1.35 | 1.98 |
| Lishui | 1.52 | 2.33 |
| Jiangning | 0.93 | 1.46 |
| Pukou | 1.03 | 1.67 |
| Luhe | 0.70 | 1.13 |
| Jurong | 0.55 | 0.84 |
| All endemic counties | 1.17 | 1.83 |
