# Supplementary material for: Ecological signature on the epidemiological dynamics of severe fever with thrombocytopenia syndrome
Source: PLoS Negl Trop Dis. 2026 Jun 8;20(6):e0014408. doi: 10.1371/journal.pntd.0014408 (PMC13245741; doi:10.1371/journal.pntd.0014408)
Supplement: S1 Text — (DOCX) [file pntd.0014408.s010.docx]

**Sensitivity analyses**

In view of the biology of tick growth and development, we assumed that tick abundance may be highly dependent on the meteorological conditions in the last month. We validated this lagged effect by assuming a 2-month association between meteorological factors and vector population dynamics. The performance of model with one-month and two-month lagged effects was quantified by the generalized cross-validation criterion (GCV), the proportion of deviation explained by model and the significant weather predictors (p < 0.05). The findings (Table S2) show that local weather conditions in the 2 months do not have a significant impact on tick abundance.

The proposed climate-based vectored transmission mechanism mirrors the nature of our eco-epidemiological model. We further sought to verify the hypothesis of the climate-based vectored transmission in the eco-epidemiological model. To this end, we proposed a “vector-free” model which alternatively assumes a direct effect of meteorological condition on SFTS transmission. This means that the transmission rate is not characterized by both vector efficacy and abundance in the alternative model; instead, a simple spline-fitted transmission rate. We show that the alternative model failed to capture the dynamics of SFTS in most counties and years (Table S3). This verifies the outperformance of the climate-based vectored transmission mechanism in characterizing complex interactions at the climate–vector–disease interface.
